# Bioethics and COVID-19: Considering the Social Determinants of Health

**DOI:** 10.3389/fmed.2022.824791

**Published:** 2022-03-22

**Authors:** Luca Valera, Rodrigo López Barreda

**Affiliations:** ^1^Bioethics Centre, School of Medicine, Pontificia Universidad Católica de Chile, Santiago de Chile, Chile; ^2^Department of Philosophy, Universidad de Valladolid, Valladolid, Spain; ^3^Department of Anesthesiology, School of Medicine, Pontificia Universidad Católica de Chile, Santiago de Chile, Chile

**Keywords:** bioethics, social determinants of health, public health ethics, shared agency, shared responsibility, vulnerability

## Abstract

In this paper, we focus on a novel bioethical approach concerning the ethical implications of the Social Determinants of Health (SDs) in the time of COVID-19, offering a fresh interpretation of our agency and responsibility in the current pandemic era. Our interpretation is grounded on the idea that our health basically depends on factors that go beyond our organism. In this sense, we stress the radical importance of circumstances to ethically assess an action, in the current pandemic context. Moreover, due the centrality of the SDs in our bioethical assessments—that implies that our health does not exclusively depend on our choices, behaviors, and lifestyle—we can affirm that we are not entirely responsible for our wellness or diseases. As health depends on economic, social, cultural, and environmental factors, we argue that the analysis of personal responsibility facing personal health status should receive further consideration. In this sense, following the “social connection model,” we stress the importance of the concept of “shared responsibility” in collective decisions: if we make many decisions collectively, we are also collectively responsible of these decisions. Furthermore, to responsibly tackle the social inequalities that are the underlying cause of disparities in health outcomes, we propose two main strategies based on the Capability Approach: 1. empowering the individuals, especially the most vulnerable ones; and 2. designing preventive policies and interventions that provides an opportunity to address the disparities moving forward. This will help us going beyond the “individualistic medical ethics paradigm” and integrating our concept of health with social factors (e.g., the SDs), based on a more relational and interdependent anthropological thought.

## Introduction

The COVID-19 pandemic has highlighted, on the one hand, the fragility of our health systems, of our way of conceiving medicine and, therefore, of our way of interpreting our social coexistence. On the other, it has emphasized the need to rethink basic anthropological issues, such as our interdependence, vulnerability, and finitude. These considerations are the result of the global reflections that have been carried out over the last 2 years. An important role, in this sense, has been played by philosophy, and, more specifically, by public health ethics and bioethics. Numerous experts have contributed to the public debate with the aim of offering interpretations and considerations on the condition of human beings in times of pandemic [e.g., (1, 2)], in order to propose principles and ethical guidelines with reference to the use of the limited medical resources available [e.g., (3, 4)] and to help in the development of policies to address the health crisis [e.g., (5, 6)].

In this regard, although the topic of justice has been at the center of the public debate, it seems to us that, when thinking at possible policies and ethical guidelines, little thought has been given to the ethical centrality of the Social Determinants of health (SDs). Although the issue of arbitrary discrimination in relation to the scarce resources available in times of pandemic has been largely addressed (5, 7), little thought has been given to the importance of rethinking ethical evaluations based on these socioeconomic factors, which are usually known as SDs. In this sense, we agree with Churchill et al. (8), when they state: “Bioethics has gone too small: it has focused primarily on bedside issues. The consequence is that it has paid scant attention to societal-level macro-issues such as the social determinants of illness and health, the structural racism that magnifies the burden of disease for people of color, and the effects of dismantling the infrastructure for public health.” In this paper we specifically focus on this novel approach with regards to the ethical implications of SDs in the time of COVID-19, offering a fresh interpretation of our agency and responsibility in the current pandemic era.

## Considering SDs for a More Adequate Clinical Interpretation

It is not only a matter of making a proper ethical (or bioethical) assessment, but also of correctly considering all the factors that influence people's health. Indeed, the recent pandemic has shown us how a different model of “health” is emerging, one that goes far beyond the simple absence of disease or the functionality of certain organs (or the whole organism). Now, it must be acknowledged that health is not only the result of individual behavior, personal predisposition, and health care provision, but also economic, social, cultural, and environmental factors (9). At the same time, a broader comprehension of the main factors that directly or indirectly affects human health—as the Systemic Clinical Risk Management (SCRM) suggests—may help the physicians to “develop a proactive approach to patient safety” (10).

In this sense, the end of the “biomedical paradigm of health” may be declared—a paradigm which is essentially individualistic and with a “pathological” approach. Conversely, emerges a more complex, systemic, multidimensional, and relational idea of human health. As Engel (11) correctly points out: “The scientific approach to disease began by focusing in a fractional-analytic way on biological (somatic) processes and ignoring the behavioral and psychosocial.” Indeed, this emerging idea of health (and, consequently, of disease) would imply a non-mechanistic interpretation of the world (and, more specifically, of the human body), inspired by von Bertalanffy's General System Theory. This paradigm would replace the biomedical one, given the evident lacks and inability to properly explain the human condition in the current era of the latter. Again Engel (11) states: “The existing biomedical model does not suffice. To provide a basis for understanding the determinants of disease and arriving at rational treatments and patterns of health care, a medical model must also take into account the patient, the social context in which he leaves, and the complementary system devised by society. […] This requires a biopsychosocial model.” This is exactly what the pandemic has shown us: we need a broader anthropological interpretation in order to understand human health.

In this regard, the COVID-19 pandemics has even shown that there is a “strong associations between crowding and airway infections, and there is reason to believe that COVID-19 is no exception” (12). Moreover, many other social factors have been related to COVID-19 outcomes, such as “poverty, physical environment (e.g., smoke exposure, homelessness), and race or ethnicity” (13). This is why a strong focus on socioeconomic status is more urgent than ever (14): our health basically depends on factors that go beyond our organism. Or better: our organism could not be isolated from its context and socioeconomic environment, as human ecology clearly highlighted (15). A significant part of this environment is: income and wealth; conditions of employment; access to health services; conditions of housing; food environment; environmental conditions; education; and safety (16–18). These are precisely the SDs, which may be understood as “the conditions in which people live their daily lives and the structural influence on these conditions that ultimately reflect the distribution of power and resources” (19). These lead to “differences in health between groups—identified by measures of socioeconomic position, occupation, education, geographical place of residence, sex, race and ethnicity, disability and intersections between groups (such as socioeconomic position and sex)” (19).

The medical and clinical relevance of these factors has been particularly evident in the current pandemic scenario, where “physical distancing measures, which are necessary to prevent the spread of COVID-19, are substantially more difficult for those with adverse social determinants and might contribute to both short-term and long-term morbidity. School closures increase food insecurity for children living in poverty who participate in school lunch programmes. Malnutrition causes substantial risk to both the physical and mental health of these children, including lowering immune response, which has the potential to increase the risk of infectious disease transmission. People or families who are homeless are at higher risk of infection during physical lockdowns especially if public spaces are closed, resulting in physical crowding that is thought to increase viral transmission and reduce access to care. Being able to physically distance has been dubbed an issue of privilege that is simply not accessible in some communities” (13). This is a faithful description of the current situation of millions of people all around the world. Obviously, this fatal scenario mostly affects certain world regions where health inequalities have been historically present: Latin-America is one of the most affected regions (2).

Nevertheless, despite the strong evidence showing the relevance of SDs in health outcomes, increased due to the current pandemic, the causal mechanisms involved are not fully elucidated, yet. Different models have been proposed, ranging from linear (20) to more complex in structure (21), including those considering geographical and temporal variables (22). Considering SDs in designing and implementing public health policies, thus, is essential to increase the effectiveness of them, as “standard compartmental epidemiological models do not adequately consider the various social determinants of health that have a direct impact on the inequalities of health outcomes and the ability of populations to effectively comply with NPIs [Non-Pharmaceutical Interventions]” (18).

## Ethical Factors and SDs

The role of SDs in health outcomes is important not only for health care practitioners and policy makers, but it is also relevant for bioethicists. As Prah Ruger (23) points out, “alongside this practical debate exists a parallel debate at the philosophical level.” In this sense, through this chapter we want to face this philosophical debate, basically focusing on the ethical implications of the new idea of health presented above.

On the one hand, as health is not only the result of individual behavior, personal predisposition, and healthcare, but it also depends on economic, social, cultural, and environmental factors, the analysis of personal responsibility facing personal health status should receive further consideration. The classical case of people requiring liver transplant due to alcoholism could be considered, then, not only as someone voluntarily engaging in an unhealthy behavior, but also as someone suffering from social conditions that render him/her prone to addictions, diminishing his/her own responsibility. The possible implications regarding other aspects, however, deserves careful consideration. “The COVID-19 pandemic has demonstrated, in profound ways, that all sectors of society and all members of society are interlinked and interdependent” (16) and this issue has not been properly explored in Western bioethical literature, yet. Our traditional attention is devoted to high-tech treatments and relies on personal autonomy as one of the most important values (together with classical “principles”), whereas African bioethicists claims that “bioethical questions related to urban poverty, drug use, immigration, occupational hazards in the workplace or environmental injustice make only rare appearances in peer-reviewed bioethics journals, course syllabi, and conferences” (24).

Even though these issues haven't been largely explored in “traditional” medical ethics, it cannot be said that this is something “absolutely new.” Just think at the “classical ethical paradigm” (25, 26). The “ordinary means or treatments” basically depend on geographical and temporal factors, cultural conditions, financial status, psychological condition of the patient, and so forth (27). Or, when ethically assessing an action, it is known that the circumstances constitute a relevant criterion to define the morality of the act itself. In this regard, the SDs may be considered as the “circumstances” in the classical ethics tradition. However, it is also illusory to claim that these aspects were of extreme importance in “classical” ethics, to the extent that we should consider, for example, that cultural circumstances can change our judgment about an action. On the other hand, it seems to us that the pandemic is inviting us to consider the radical importance of circumstances—along with the other factors that determine the morality of the human act—when ethically assessing an action. These considerations do not necessarily imply forms of “circumstantialism,” casuistry, or relativism, evidently, but only invite us to more comprehensive moral evaluations.

A broader ethical consideration of health is more than urgent nowadays, indeed. On the one hand, if our health does not exclusively depend on our choices, behaviors, and lifestyle, we can affirm that we are not entirely responsible for our wellness or diseases. On the other, if we are interdependent and mutually vulnerable—the new paradigm of “One Health” (28) basically expresses this fact—our health choices may affect other lifestyles and health. Some lessons can be learned from geriatric approaches that have considered factors beyond clinical issues, including social and environmental, to assess complex constructs, such as frailty (29). A good example of this multidimensional approach is the treatment of illnesses associated to loneliness in elderly people. Indeed, investigations argue that this phenomenon may predict functional decline and death in elderly population (30, 31). In this regard, a successful public health initiative should reduce social disconnection (29) by “facilitating participation in community activities, thereby protecting against the development of affective disorders” (32).

These considerations imply a different form of responsibility: where does our responsibility begin and end? Does it still make sense to speak simply of individual responsibility or is it better to reframe it as “shared responsibility”? As we said, as health is not only the result of individual behavior, personal predisposition, and healthcare, but also depends on economic, social, cultural, and environmental factors, the analysis of personal responsibility facing personal health status should receive further consideration. In this regard, the “social connection model” proposed by Young (33) may provide interesting insights to analyze individual and social responsibility facing collectively determined facts. She argues: “Our responsibility derives from belonging together with others in a system of interdependent processes of cooperation and competition through which we seek benefits and aim to realize projects” (33). This fact doesn't imply the inexistence or irrelevance of personal responsibility, though. It is just a different kind of approach to the issue of responsibility and agency: it focuses on humanity as a moral agent, from which a new sense of responsibility may emerge, as suggested by Jonas (34). In this sense, the concept of “shared responsibility” in collective decisions (35) is the counterpart of the idea of “shared agency” (36, 37): if we make –implicitly or not—many decisions collectively, we are also collectively responsible of these decisions.

Obviously, the degree of responsibility of the individual in collective decisions is quite distinct from that of individual decisions. In fact, the two types of actions have practical effects (even at the level of evaluation) on distinct areas of life: if individual actions concern the “moral” life of the individual (and ethics is the discipline that evaluates them), collective actions relate to the “public” life of persons (and public policies are its test bench). This last consideration about shared responsibility allows us, therefore, to examine and address public health policies in times of pandemic, with particular attention to SDs.

## What Should We Do? Morality and Policies

The aforementioned issues for clinical practice and philosophy revealed by the COVID-19 pandemic constitute a call for action. As Burström and Tao (12) claim, “an important starting point is increasing knowledge and awareness of the underlying mechanisms; studies are needed to understand how the disease strikes and by which pathways it impacts certain population groups more adversely—taking lessons from previous disease outbreaks.” This information will provide us with the required knowledge to design and implement policies that would effectively decrease health disparities, as those shown by COVID-19 (13). Additionally, healthcare access has been shown to be a determinant on explaining these disparities and it has become urgent to implement “laws and policies to ensure access to healthcare services is based on medical need rather than on ability to pay or social status and that services are tailored to recipients' cultural, linguistic, and religious requirements” (38). Echoing the statement by Takian et al. (39), we argue that “viruses do not discriminate, nor should health systems.”

Following our previous consideration, anyway, in order to face this problem our actions should go beyond health sector and involve the society as a whole. Social inequalities are the underlying cause of disparities in health outcomes. Therefore, “the pandemic has highlighted the unequal distribution of power and resources, and people are also using this moment to challenge these inequalities anew” (38).

A suitable framework to address this challenge, then, could be represented by the Capability Approach (CA), proposed by Amartya Sen. This perspective “emphasizes the importance of human agency—i.e., people's ability to live a life they value. It underscores that agency is essential for both individual and collective action and is critical for changing policy, norms, and social commitments. Reducing social inequalities in health therefore requires more than ‘flattening the socioeconomic gradient”' (23). CA, then, calls for new forms of social commitment, understanding democracy as something more than representative governments where citizens have the right to vote, but a society in which empowered persons have multiple ways to participate in public deliberation and decision making (40). This may be a daunting challenge, however, “interventions to tackle systematically reproduced conditions of vulnerability would contribute toward a fairer and more sustainable world” (38). To do so, we may follow two main strategies: 1. Empowering the individuals, especially the most vulnerable ones (23)—e.g., developing, effective communication (41); and 2. Designing preventive policies and interventions that provides an opportunity to address the disparities moving forward (13), starting from an increasing knowledge and awareness of the underlying mechanisms of health inequalities (12). In this regard, bioethics can provide arguments to challenge traditional development assessment approaches, such as the Gross Domestic Product (GDP), and pay attention to multidimensional and more comprehensive strategies, such as the Human Development Index. While the second strategy is more common and more frequently addressed by the public discourse, the first one is less used and applied, since it represents a long-range challenge. This first strategy may represent the new educational challenge emerging from this time of pandemic that can drive changes to future national and international policies and guidelines.

These two strategies are not mutually exclusive, obviously. Furthermore, we may state that they are complementary since they address the same problems and concerns (the emerging of SDs and the pandemic) but starting from different points of view (i.e., top-down and bottom-up). These two have same aim: to improve the conditions “under which individuals are free to choose healthier life strategies and conditions for themselves and for future generations” (23). The CA has a preeminent role, thus: it focuses on “the empowerment of individuals to be active agents of change in their own terms –both at the individual and collective level” (23).

## Conclusions: Bioethics May Go far

The time of pandemic is, basically, a time of changes. We changed our behaviors, our lifestyle, our worldview, our perception of the future, our concept of health and illness, and so forth. We may add: it is time to change our bioethical view, too.

It is time to integrate our “classical” view of ethics with the new evidence that are currently emerging. In this sense, the history of bioethics may help us. The classic contention (42–44) between Wisconsin (i.e., Potter's global bioethics) and Georgetown (i.e., Hellegers and Callahan's medical ethics) doesn't make sense anymore. It is time to go beyond the “individualistic medical ethics paradigm” to develop theoretical bridges toward public health ethics, environmental ethics, and global policies issues (45). This may help us integrating our concept of health as a “biological issue” with social factors (e.g., the SDs), based on a more relational and interdependent anthropological thought. This is an urgent step we should take in bioethical inquiries, in addition to assume an active role in policy making, advocating for a fair balance between social interdependence and individual autonomy.

On the one side, thus, we may think, together with Churchill et al. (8), that “bioethics has gone too small,” if we consider only the Georgetown paradigm (46), which seems to be too narrow for current pandemic concerns. On the other, redeeming Potter's (47) idea of bioethics, this novel form of considering SDs as the circumstances of the action may help developing the bridge between public health ethics, bioethics, and environmental ethics (45). In brief, a bridge to the future. Bioethics may, thus, go far.

## Data Availability Statement

The original contributions presented in the study are included in the article/supplementary material, further inquiries can be directed to the corresponding author/s.

## Author Contributions

All authors listed have made a substantial, direct, and intellectual contribution to the work and approved it for publication.

## Conflict of Interest

The authors declare that the research was conducted in the absence of any commercial or financial relationships that could be construed as a potential conflict of interest.

## Publisher's Note

All claims expressed in this article are solely those of the authors and do not necessarily represent those of their affiliated organizations, or those of the publisher, the editors and the reviewers. Any product that may be evaluated in this article, or claim that may be made by its manufacturer, is not guaranteed or endorsed by the publisher.
